# Harnessing extracellular vesicles to tame inflammation: a new strategy for atherosclerosis therapy

**DOI:** 10.3389/fimmu.2025.1625958

**Published:** 2025-06-18

**Authors:** Jiawei Li, Tianliang Wen, Xiaoran Li, Ruoyao Cheng, Junyi Shen, Xin Wang, Zhaoqi Guo, Zhengjie Teng, Lin Yi, Fan Zhang

**Affiliations:** ^1^ School of Traditional Chinese and Western Medicine, Gansu University of Chinese Medicine, Lanzhou, Gansu, China; ^2^ First School of Clinical Medical, Gansu University of Chinese Medicine, Lanzhou, Gansu, China; ^3^ Department of Anesthesiology and Surgery, Affiliated Hospital of Gansu University of Chinese Medicine, Lanzhou, Gansu, China; ^4^ Department of Cardiovascular Medicine, Affiliated Hospital of Gansu University of Chinese Medicine, Lanzhou, Gansu, China; ^5^ Chronic Disease Laboratory, Gansu University of Traditional Chinese Medicine, Lanzhou, Gansu, China; ^6^ Geriatrics Department, Affiliated Hospital of Gansu University of Chinese Medicine, Lanzhou, Gansu, China

**Keywords:** atherosclerosis, extracellular vesicles, exosomes, microvesicles, intercellular communication, biomarkers, nanotherapy

## Abstract

Atherosclerosis (AS) is a chronic inflammatory disease driven by immune dysregulation and vascular inflammation. Extracellular vesicles (EVs) play pivotal roles in intercellular communication, modulating immune responses and inflammatory cascades during AS progression. EVs derived from endothelial cells, macrophages, vascular smooth muscle cells, and platelets transport bioactive molecules (e.g., miRNAs, cytokines) that regulate endothelial dysfunction, macrophage polarization, and plaque instability. Pro-inflammatory EVs exacerbate oxidative stress, foam cell formation, and neutrophil extracellular trap (NET) release, while anti-inflammatory EVs from mesenchymal stem cells or engineered sources attenuate disease by promoting M2 macrophage polarization and suppressing NF-κB signaling. This review highlights the dual roles of EVs in AS immunopathology and their therapeutic potential as biomarkers or nanocarriers for targeted anti-inflammatory interventions. Understanding EV-mediated immune crosstalk may unveil novel strategies for atherosclerosis management.

## 1 Introduction

Atherosclerosis (AS), a chronic inflammatory vascular pathology characterized by lipid deposition, endothelial dysfunction, and plaque formation, persists as the principal contributor to global cardiovascular and cerebrovascular morbidity and mortality (1). The disease progression involves a complex interplay of endothelial activation, vascular smooth muscle cell (VSMC) phenotypic modulation, and sustained immune-inflammatory responses within the arterial wall, culminating in luminal stenosis and end-organ ischemia (2). Elucidating these mechanistic underpinnings is critical for developing targeted therapeutic interventions.

Extracellular vesicles (EVs), comprising exosomes (30–150 nm) derived from endosomal multivesicular bodies and microvesicles (50–1,000 nm) shed via plasma membrane budding, have emerged as pivotal mediators in AS pathogenesis (3, 4). While microvesicles retain surface markers reflective of parental cells, exosomes encapsulate dynamic molecular cargoes that mirror cellular states, rendering them promising biomarkers and therapeutic vectors (4). In AS, EVs orchestrate oxidative stress amplification, inflammatory cascades, VSMC calcification, and plaque destabilization through intercellular communication (5, 6). Their bidirectional roles—both aggravating and mitigating disease processes—highlight their potential for diagnostic exploitation and precision therapeutics (7). This review systematically summarizes EV contributions from diverse cellular origins, including endothelial cells, macrophages, and VSMCs, and their translational implications in AS management.

## 2 Roles of EVs from distinct cellular origins in AS

Extracellular vesicles (EVs) associated with AS are predominantly secreted by endothelial cells (ECs), VSMCs, macrophages, and platelets, collectively accounting for the majority of circulating EVs (8). EVs originating from diverse cell types are released into the circulation and serve as critical mediators for the long-range transfer of bioactive molecules (**Table 1**).

**Table 1:** Summary of the contents carried by EVs from different cell sources and their biological functions in AS.

EV Source	Key Cargo	Target Cell/Pathway	Functional Role in AS
Endothelial Cells (ECs)	miR-206, miR-143/145, miR-155, MALAT1	VSMCs (ARF6/NCX1 axis), macrophages (NF-κB)	Modulates VSMC phenotypic switching (contractile→synthetic); promotes M1 macrophage polarization; induces NET formation. Anti-atherogenic effects via KLF2-miR-143/145 axis.
Vascular Smooth Muscle Cells (VSMCs)	miR-155, miR-204/211, miR-222	ECs (CD36), VSMCs (osteogenic differentiation)	Promotes EC injury and plaque calcification; exacerbates neointimal hyperplasia. Melatonin-treated EVs attenuate calcification via miR-204/211.
Macrophages	miR-19b-3p, miR-146a, miR-185-3p, lncRNA GAS5	VSMCs (JAZF1), ECs (VCAM-1), neutrophils	Drives VSMC migration/apoptosis; enhances EC inflammation and oxidative stress; promotes foam cell formation and plaque instability.
Platelets	miR-223, miR-339, miR-21	Macrophages (CD36), ECs (ICAM-1)	Pro-thrombotic effects via TF exposure; anti-atherogenic roles by suppressing ox-LDL uptake and EC inflammation.
Other Sources	miR-1 (hepatocytes), miR-326-3p (alveolar cells), LIPCAR (foam cells)	ECs (NF-κB/VCAM1), VSMCs (proliferation)	Exacerbates endothelial inflammation (NAFLD-EVs); promotes VSMC migration (foam cell-EVs). MSC-EVs (miR-125b, miR-512-3p) attenuate AS.

NET, neutrophil extracellular trap; ox-LDL, oxidized low-density lipoprotein; KLF2, Krüppel-like factor 2; TF, tissue factor; NAFLD, non-alcoholic fatty liver disease; MSC, mesenchymal stem cell. The symbol"→" means "transition to" or "switch to.".

### 2.1 Endothelial cell-derived extracellular vesicles in atherosclerosis

ECs play a central role in regulating inflammation, coagulation, vascular tone, and endothelial permeability. Endothelial dysfunction triggers the massive release of EVs, which transfer molecular signals to recipient cells such as ECs, VSMCs, and macrophages, thereby modifying their function and contributing to AS pathogenesis. EC-derived EVs can reduce nitric oxide (NO) production and alter vascular tone and endothelial function by modulating NO phosphorylation or promoting local oxidative stress (9). In a diabetic murine model of AS, endothelial progenitor cell (EPC)-derived EVs significantly ameliorated endothelial dysfunction and reduced AS plaque burden and inflammatory cytokine expression (10). The phenotypic switching of VSMCs from a contractile to a synthetic state represents a critical step in AS pathology. EC-derived EVs can modulate this transition via their molecular cargo; for example, EVs enriched in miR-206 target the ARF6/NCX1 axis to preserve the contractile phenotype of VSMCs (11). EVs from KLF2-overexpressing ECs induce an anti-atherogenic VSMC phenotype through the miR-143/145 axis (12). Conversely, ECs activated by CD137 signaling exhibit reduced TET2 expression in their EVs, promoting VSMC phenotypic switching and neointima formation (13). Under pathological stimuli such as oxidative stress, EC-derived EVs transfer miR-92a-3p to VSMCs, enhancing proliferation and migration while exacerbating vascular inflammation (14).

Beyond modulating VSMC phenotypes, EC-derived EVs exert pro- or anti-inflammatory effects on macrophages under various stimuli. EVs from KLF2-overexpressing ECs exhibit reduced levels of miR-155, thereby enhancing immunomodulatory responses and suppressing monocyte activation, ultimately attenuating AS lesion formation. In contrast, ox-LDL-stimulated EC-derived EVs are enriched in miR-155, which reprogram macrophages from an anti-inflammatory M2 to a pro-inflammatory M1 phenotype (15). These EVs may also carry metastasis-associated lung adenocarcinoma transcript 1 (MALAT1), promoting neutrophil extracellular trap (NET) formation and aggravating AS (16).

### 2.2 Vascular smooth muscle cell-derived extracellular vesicles in atherosclerosis

The proliferation, phenotypic modulation, apoptosis, and calcification of VSMCs are intimately linked to AS development. Through EV-mediated communication, ECs and VSMCs regulate vascular homeostasis and AS progression. Under pathological conditions, synthetic VSMCs secrete EVs that induce EC migration and angiogenesis, contributing to plaque formation and vascular calcification (17). EVs from KLF5-overexpressing VSMCs are enriched in miR-155 and induce endothelial injury and AS via intercellular transfer (18). Proliferative VSMCs release excessive EVs that deposit within the vasculature, facilitating mineralization and vascular calcification. miRNAs are the primary functional cargo of these EVs; for instance, melatonin-treated VSMC-derived EVs carrying miR-204 and miR-211 attenuate osteogenic differentiation of recipient VSMCs, ameliorating vascular calcification and aging (19).

### 2.3 Macrophage-derived extracellular vesicles in atherosclerosis

Macrophages and monocytes residing in the subendothelial space participate in all stages of AS, from endothelial dysfunction to lesion expansion and plaque formation. Macrophage-derived EVs enriched in miR-19b-3p promote VSMC migration via suppression of zinc finger gene JAZF1, thereby accelerating AS (20). Similarly, EVs containing miR-503-5p affect both ECs and VSMCs, enhancing plaque formation (21). Pro-inflammatory M1 macrophage-derived EVs carrying miR-222 stimulate VSMC proliferation and migration, exacerbating neointimal hyperplasia in mice (22). Other studies report that EVs from M1 macrophages are rich in miR-185-3p, which enhance EC adhesion, oxidative stress, and pro-inflammatory cytokine release while inhibiting proliferation and promoting endothelial apoptosis, thus worsening AS (23). EV-derived miR-146a from AS-associated macrophages promotes macrophage retention and impaired migration, thereby contributing to lesion progression (23, 24). Nicotine-stimulated macrophage-derived EVs mediate VSMC proliferation and migration through the miR-21-3p/PTEN axis (25). High-glucose conditions induce macrophages to release EVs enriched in miR-486-5p, which promote cell proliferation and hematopoiesis, thereby advancing AS (26). Monocyte-derived EVs under ox-LDL stimulation serve multiple roles. For example, they carry lncRNA GAS5, which regulates apoptosis in macrophages and ECs (27), and miR-186-5p, which inactivates the SHIP2-mediated PI3K/AKT/mTOR pathway to enhance VSMC viability and invasiveness (28). Moreover, monocyte-derived EVs can carry miR-146a to promote oxidative stress, neutrophil extracellular trap formation, and AS progression (29).

### 2.4 Extracellular vesicles from other cell types in atherosclerosis

Platelets are key regulators of vascular homeostasis through interactions with circulating blood cells and the vessel wall. Platelet-derived EVs interact with diverse cell types, activating ECs and recruiting monocytes, thereby contributing to AS (30). These EVs enhance ox-LDL uptake and inflammatory cytokine release in macrophages, promoting foam cell formation (31). EVs from monocyte-platelet aggregates stimulated with tumor necrosis factor-α (TNF-α) exhibit pro-inflammatory effects on ECs and atherosclerotic plaques (32). Thrombin-activated platelet EVs are enriched in miR-223, miR-339, miR-21, and miR-25-3p, which suppress VSMC proliferation, promote apoptosis, reduce ox-LDL-induced EC inflammation and lipid accumulation, and inhibit AS progression (33). Hepatocyte-derived EVs under non-alcoholic fatty liver disease conditions transport miR-1, suppress KLF4, and activate the NF-κB pathway, leading to endothelial inflammation and promoting AS (34). EVs from PM2.5-exposed alveolar epithelial cells, rich in TNF-α and miR-326-3p, activate the IκBα-NF-κB-VCAM1 axis, driving AS progression (35). Insulin-resistant adipocyte-derived exosomes promote AS plaque rupture by inducing angiogenesis (36). Foam cell-derived EVs containing lncRNA LIPCAR promote VSMC proliferation and migration, exacerbating AS (14). Mesenchymal stem cell (MSC)-derived exosomal miR-512-3p modulates Keap1 and prevents ox-LDL-induced endothelial dysfunction (37). MSC-derived EVs also deliver miR-125b to VSMCs, suppressing proliferation and migration, and reducing neointimal growth (14). Additionally, these EVs inhibit AS plaque expansion by reducing macrophage infiltration (38). EVs released from AS plaques may transport pro-inflammatory factors such as miR-23a-3p, facilitating endothelial inflammation and macrophage infiltration, and potentially spreading AS to distant vascular sites (39).

### 2.5 Uptake mechanisms of extracellular vesicles by recipient cells

To exert their biological effects, EVs are internalized by recipient cells (40). Various uptake mechanisms have been identified, including clathrin-dependent endocytosis, caveolin-mediated endocytosis, macropinocytosis, phagocytosis, and membrane fusion (41–43). The pathway utilized depends on the EV subtype, cellular origin, membrane composition, and the target cell type. For instance, endothelial cells internalize platelet-derived EVs primarily via endocytosis (44). Conversely, macrophages often uptake EVs through phagocytosis or macropinocytosis, pathways suited for larger vesicles such as microvesicles (45). Lipid raft-mediated endocytosis is commonly employed for exosome internalization in VSMCs (46, 47). Additionally, in certain conditions, direct fusion of EVs with the plasma membrane may occur, allowing for rapid delivery of cargo such as miRNAs and proteins into the cytosol (48). Surface molecules including tetraspanins (CD9, CD63), integrins, heparan sulfate proteoglycans (HSPGs), and phosphatidylserine receptors play crucial roles in mediating cell-specific EV uptake (49, 50). Notably, the expression patterns of these receptors vary in disease states, influencing EV-target cell specificity during atherosclerosis progression (51). Understanding these uptake pathways not only provides insights into EV biology but also offers opportunities to enhance the design of targeted EV-based nanotherapeutics.

## 3 Roles of extracellular vesicles in different stages of atherosclerosis

Extracellular vesicles (EVs) play critical roles across various stages of atherosclerosis (AS) development, including the initiation of atherosclerotic lesions, vascular calcification, progression of unstable plaques, and thrombosis following plaque rupture.

### 3.1 EVs in the initiation of atherosclerotic lesions

Atherosclerosis is a chronic inflammatory disease initiated by sustained endothelial injury, where endothelial dysfunction constitutes an early pathological hallmark. EVs released under pathological conditions can facilitate the infiltration of oxidized low-density lipoprotein (ox-LDL) into endothelial cells (ECs), promoting early lesion development (52). Beyond serving as biomarkers of endothelial dysfunction, EVs exert direct biological effects on ECs, playing a pivotal role in promoting cellular dysregulation (53). Circulating EVs can transfer arginase to ECs, resulting in reduced nitric oxide (NO) production and impaired vasodilatory function. Nicotine-induced monocyte-derived EVs enriched in miR-155 exacerbate EC dysfunction and accelerate AS progression (54). Inflammation is a major driver of EV release during AS pathogenesis. EVs containing miR-126-5p and miR-212-3p have been shown to activate monocytes in early radiation-induced vascular inflammation associated with AS. EC-derived EVs transfer a functional mixture of inflammatory mediators to target cells and regulate monocyte activation by modulating their miRNA cargo. For instance, EC-derived EVs transfer miR-10a into monocytes to inhibit inflammation via the NF-κB signaling pathway (55). EVs secreted by TNF-α-stimulated ECs carry inflammatory markers such as CCL-2, IL-6, IL-8, and ICAM-1, promoting monocyte adhesion and migration into plaques (56). Additionally, platelet-derived EVs activate macrophages, enhancing ox-LDL phagocytosis and inflammatory cytokine release (31).

### 3.2 EVs in atherosclerotic progression

During AS progression, lipids accumulate in the subendothelial space of injured arteries and are oxidatively modified and taken up by macrophages in the arterial wall. EVs derived from various cell types promote foam cell formation by enhancing lipid and cholesterol accumulation in macrophages. Upon EV clearance, macrophages and foam cells may undergo programmed cell death, contributing to extracellular lipid core expansion. Exosomes secreted by T cells within atherosclerotic plaques promote cholesterol accumulation and pro-inflammatory cytokine production in monocytes and macrophages, thereby accelerating AS development (57). Phenotypic switching, proliferation, migration, and secretion of vascular smooth muscle cells (VSMCs) are hallmarks of advanced AS (12). EVs are involved in these processes by modulating VSMC behavior. For example, EVs derived from macrophages or ox-LDL-treated macrophages can promote VSMC proliferation and migration, contributing to plaque progression (20).

### 3.3 EVs and vascular calcification

Vascular calcification is a key feature of advanced atherosclerotic plaques. While microcalcification increases the risk of plaque rupture, macrocalcification may contribute to plaque stability. Coronary intimal calcification can occur across all stages of AS, from early intimal thickening to late lesions with large necrotic cores. EVs secreted by ECs, VSMCs, or macrophages have been identified within atherosclerotic plaques. Pro-inflammatory macrophage-derived matrix vesicles (MVs) promote microcalcification via enriched expression of phosphatidylserine (PS), S100A9, and Annexin V (Anx5), forming a PS–Anx5–S100A9 membrane complex that serves as a nucleation site for hydroxyapatite crystals (58).

Calcification frequently occurs alongside apoptosis of VSMCs and macrophages, coupled with MV release and expression of osteogenic markers in the vessel wall. The degree of calcification correlates with plaque burden, and microcalcifications within thin fibrous caps may alter local mechanical stress and promote rupture. Plaque calcification is thought to occur through four interrelated mechanisms. First, apoptotic bodies and necrotic debris originating from macrophages and vascular smooth muscle cells (VSMCs) act as nucleation sites for calcium phosphate crystal formation. Second, circulating nucleation complexes derived from bone remodeling processes or MVs released by VSMCs and macrophages initiate the crystallization of amorphous calcium phosphate. Third, when phosphate concentrations exceed a critical threshold, minerals within MVs are deposited onto osteogenic collagen fibers. Finally, a reduction in circulating and tissue-secreted calcification inhibitors facilitates the reorganization of amorphous calcium phosphate into hydroxyapatite, thereby promoting the osteogenic differentiation of VSMCs (59).

### 3.4 EVs in plaque instability and thrombosis following rupture

**Figure 1:**
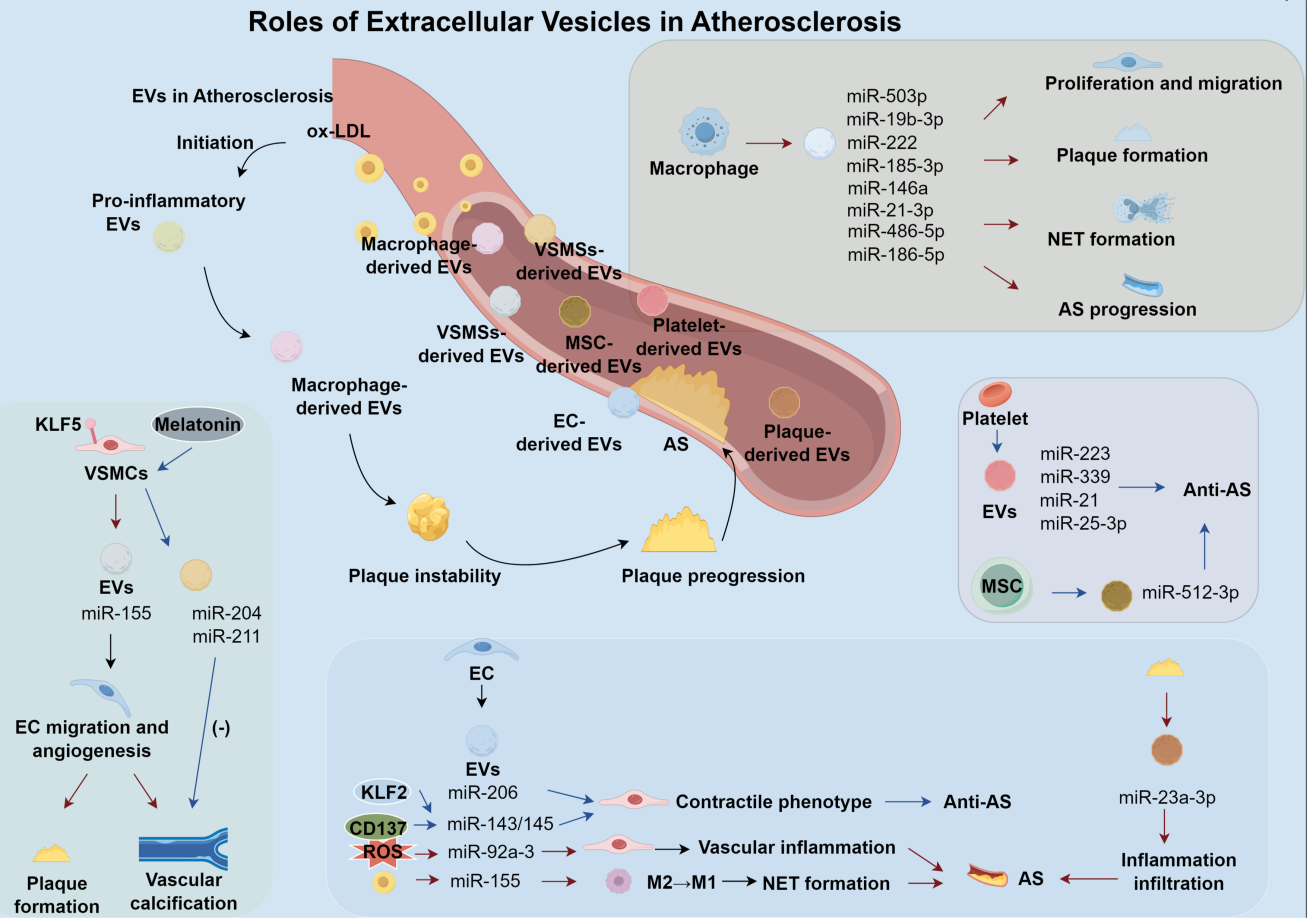
Role of extracellular vesicles in atherosclerosis.

EVs are implicated in the pathophysiological processes underlying plaque instability and thrombosis. Advanced plaque characterization reveals the accumulation of thrombogenic EVs within the necrotic core. Atherosclerotic plaques contain significantly higher EV levels than plasma, and these plaque-associated EVs exhibit enhanced prothrombotic potential (60). MVs within fibrous caps may destabilize plaques by altering the extracellular matrix of connective tissues, leading to rupture. Upon rupture, thrombogenic EVs are exposed, triggering platelet activation and thrombus formation at the lesion site (61). EVs can initiate the coagulation cascade through two distinct mechanisms. They expose tissue factor on their surface, especially in EVs derived from monocytes, which triggers thrombin generation and fibrin clot formation (60). Additionally, EVs present phosphatidylserine (PS) on their outer membrane, providing a catalytic surface that concentrates and activates coagulation factors VII and VIIa (62). While EVs predominantly promote thrombosis, certain subtypes may also exert antithrombotic effects. For example, platelet-derived EVs have been shown to reduce CD36-dependent lipid uptake in macrophages and suppress platelet thrombus formation, thereby mitigating AS-related thrombosis (63) (**Figure 1**).

## 4 Therapeutic applications of extracellular vesicles in atherosclerosis

Exosomes derived from various cell types have been shown to contribute to the pathogenesis of atherosclerosis (AS); however, accumulating evidence suggests that extracellular vesicles (EVs) derived from mesenchymal stem cells (MSCs), endothelial progenitor cells (EPCs), bone marrow-derived macrophages, and platelets exert beneficial effects in ameliorating AS. MSCs-derived EVs have demonstrated therapeutic potential in the context of AS. Notably, EVs secreted by human umbilical cord MSCs (HUCMSCs) exhibit high expression levels of miR-100-5p, which attenuates the migration of eosinophils and inflammatory responses via inhibition of the Wnt signaling pathway, thereby suppressing the progression of atherosclerosis (64). EVs from murine bone marrow MSCs (BMSCs), enriched in miR-512-3p, have been reported to prevent AS by inhibiting endothelial cell (EC) apoptosis and inflammatory activation (35). In addition, MSC-derived EVs reduce atherosclerotic plaque size and macrophage infiltration, while promoting M2 macrophage polarization in apolipoprotein E-deficient (ApoE^−/−^) mice fed a high-fat diet, indicating their therapeutic applicability in AS (37).

Administration of EPC-derived EVs into diabetic AS mouse models markedly reduces atherosclerotic lesion formation, oxidative stress, and inflammation, while improving endothelial-dependent vasomotor dysfunction (8). These EVs, carrying miR-199a-3p, mitigate vascular endothelial injury and delay AS onset (63). EVs released from bone marrow-derived macrophages are enriched in miR-99a, miR-146b, and miR-378a, which collectively suppress inflammatory signaling by targeting NF-κB and TNF-α pathways. These vesicles also promote polarization of recipient macrophages toward an M2 anti-inflammatory phenotype, highlighting their potential as therapeutic agents for AS and other chronic inflammatory disorders (65, 66). EVs secreted by thrombin-activated platelets also exhibit anti-atherogenic properties. These platelet-derived EVs are enriched with anti-inflammatory microRNAs, including miR-223, miR-339, and miR-21 (67). Specifically, miR-223 targets ICAM-1, suppressing endothelial inflammation and thereby preventing AS. Similarly, miR-30 inhibits ox-LDL-induced EC inflammation and systemic inflammatory responses in AS murine models via modulation of the NF-κB signaling cascade (68). Beyond their direct therapeutic role, EVs also serve as promising nanocarriers for atherosclerosis-related therapeutics, capable of delivering bioactive molecules such as microRNAs, mRNAs, or proteins. Through cargo engineering or surface ligand modification to enhance binding specificity to target cells, EVs can be repurposed as precision delivery vehicles. For instance, curcumin and demethoxycurcumin alter the expression profiles of miR-125a-3p, miR-92a-3p, miR-126-5p, and miR-143-3p within EC-derived EVs, ultimately reducing proliferation and migration of VSMCs and decreasing lipid accumulation in VSMCs (69).

Furthermore, EVs secreted by M2 macrophages engineered with 5-aminolevulinic acid hexyl ester hydrochloride (HAL) exhibit active targeting capabilities to AS lesions. Once localized, the encapsulated HAL undergoes intrinsic biosynthesis and heme metabolism, generating anti-inflammatory nitric oxide (NO) and bilirubin. This dual action not only enhances the anti-inflammatory effect but also significantly alleviates AS progression (70). It is important to note that the heterogeneity of EVs and the lack of standardized isolation and characterization protocols may influence the interpretation and reproducibility of experimental findings. While this review centers on the functional and therapeutic roles of EVs in atherosclerosis, methodological aspects, though highly relevant, are beyond its scope and warrant dedicated discussion elsewhere.

## 5 Conclusion

Atherosclerosis is a complex inflammatory disease driven by dysregulated immune responses and chronic vascular inflammation. Extracellular vesicles (EVs) have emerged as critical mediators of intercellular communication, facilitating crosstalk among endothelial cells, immune cells, and vascular smooth muscle cells. Through their diverse cargo, including miRNAs, cytokines, and signaling proteins, EVs regulate key processes such as endothelial dysfunction, macrophage polarization, neutrophil extracellular trap formation, and plaque destabilization. Pro-inflammatory EVs exacerbate disease progression by promoting oxidative stress, foam cell formation, and pro-inflammatory cytokine release, while anti-inflammatory EVs derived from mesenchymal stem cells or engineered sources can attenuate atherosclerosis by suppressing NF-κB signaling, enhancing M2 macrophage polarization, and improving endothelial repair. Understanding the dual roles of EVs in immune activation and resolution provides new insights into atherosclerosis pathogenesis and highlights their potential as both diagnostic biomarkers and therapeutic vehicles.

Despite these advances, significant challenges remain in translating EV-based research into clinical applications. Standardizing EV isolation methods, improving targeting specificity, and scaling up production are critical steps for therapeutic development. Another concern is the potential for off-target effects, as systemically administered EVs can be non-specifically taken up by unintended cell populations. Future studies should focus on elucidating EV heterogeneity across disease stages, optimizing engineered EVs for precise immunomodulation, and validating their efficacy in preclinical models and human trials. By harnessing EVs’ natural ability to regulate inflammation and immunity, researchers may unlock novel strategies for atherosclerosis treatment, shifting the paradigm from generalized anti-inflammatory
